# Some of the More Prevalent Diseases Affecting Animals

**Published:** 1898-01

**Authors:** A. W. Clement

**Affiliations:** State Veterinarian, Baltimore, Md.


### SOME OF THE MORE PREVALENT DISEASES AFFECTING ANIMALS.[1]

By A. W. Clement, V.S.,
STATE VETERINARIAN, BALTIMORE, MD.

The subject of this paper is a somewhat long and wearying one, but I shall confine my remarks to the discussion of some of the diseases which are communicable from man to animals, or vice versa, with one exception—that of the disease affecting horses, which has of late been epidemic in some parts of our State. The diseases which are transmissible from man to animals and from animals to man are very interesting, and are of importance to the community. They are diseases to which a very considerable amount of study has been devoted, which has been attended with a very great amount of good. What Koch has done for tuberculosis, and what Pasteur has done for rabies, will long be remembered as the very bulwarks of modern medicine.

The question of tuberculosis alone is of great importance, both as a commercial question and as one affecting the life and comfort of a very considerable portion of our community. A disease which causes the death of one in five, or one in seven, of our population is no laughing matter, I can assure you—a disease which is causing to-day more money to be spent upon it than are all diseases to which humanity is susceptible ; a disease which is preventable to a great extent, and one to which science is devoting more attention to-day than it is to any disease affecting man or animals. It is an infectious disease ; there is no doubt about this, as all of you who are physicians, and as many of you who are not physicians, know. It is not only an infectious disease, but the means of infection have been thoroughly studied. The organism causing the disease was isolated by Koch, and its properties are well known to all men who work in laboratories. They know that the organism can be detected in organs affected ; that it can be grown outside of the body in proper nutriment, and that if inoculated into a healthy animal that animal will probably become tuberculous. Knowing these things as we do, it behooves mankind to do all in its power to prevent the poison from accumulating in the bodies of individuals. Just how to do this is a question which has agitated and is now agitating the minds of scientific investigators. Whether by curative agents, or rather by antitoxic agents, to produce a condition of body which is antagonistic to the growth of the tubercle bacilli ; whether by bettering the conditions of humanity so as to make them stronger, better able to withstand the attacks of the tubercle bacilli; or whether to enforce a quarantine upon people suffering from the disease and against all food-products which may in any way transmit the disease. These, I say, are the main lines of work of the sanitarian to-day, and are more or less fruitful in their results.

Now I do not wish to be called an extremist ; I do not wish to be an alarmist ; but I do believe that the question of our milk-supply has very much to do with the disease in children and in adults who drink much of it, and who are in any way delicate. Tubercle bacilli have repeatedly been found in the milk of cows which were visibly affected, and also in the milk of cows which did not show any of the characteristic symptoms of the disease. All milk from cattle which react to the tuberculin-test should be condemned and the cattle slaughtered. This may seem a harsh statement, and one which the dairymen of the State will not relish so well ; but it is nevertheless a fact. It will cost a lot of money, and who is to pay it ? Is the State to do it, or are the producers themselves to do it directly ? I think the latter. I do not believe the State can do it directly. Some States have expended large sums of money, and are to-day expending large sums of money, to eradicate the disease from cattle, and I am much of the opinion that it is, to a large extent, a waste of money. Government supervision of such work is always more expensive than the same work undertaken and carried out as a private enterprise. Still, the work ought to be done, and ought to be done as long as tuberculosis exists, and better have the State do it than not to have it done at all. While I say that the State ought not to do it, I do not mean by any means that the State ought not to have anything to do with it. I do think that the State, through its proper office, should have control of the work, but that it should be paid for by the producer himself.

Certain men in the medical and veterinary profession are to be trusted, and more are not. Certain ones will give an honest opinion, while more will give an opinion based upon the amount of money involved in the transaction, and the one opinion may have just as much weight with the public as the other. Before he is allowed to inspect dairy-stables and give certificates, the inspector should first receive the indorsement of the Live-stock Sanitary Board, and this indorsement should be evidence to the public of his fitness for the position. What the public need is to be educated up to a pure milk-supply, and a pure milk-supply can only be assured where the stables are under the constant supervision of a veterinarian, and of an honest veterinarian, too. Why would it not be well for the larger of our milk-dairies to unite and form a syndicate or trust for the production of pure milk? to have their stables inspected and their cattle tested by a competent man, and advertise this fact to the public? How long would it be before other dairies would have to get in line or close up shop? If only a few could be gotten to do this at first, what an educator it would be to the public. Objection will be raised to the cost, but how much less the cost would be than if attempted by the State, and how much greater would be the result? The only way to insure pure milk is by inspection of the dairy-stables, not by the inspection of milk after it arrives at its destination in the city, though that would have to be done, too, to provide against the practice of some dairies watering the milk to increase the amount.

Tuberculosis is prevalent in the State of Maryland among its cattle; this has been proven by a system of inspection inaugurated by the present Live-stock Sanitary Board, which shows that $1\frac{47}{100}$ per cent. have tuberculosis to the naked eye. That is, so many were diseased so badly as to be evident without further examination. That this number would be increased many times by the use of tuberculin has been demonstrated in my own private practice and from the statistics of other States. I have repeatedly seen cattle which were apparently healthy react to the test, and when slaughtered found tuberculous. I have in my own mind one herd belonging to a gentleman in which one tuberculous animal called attention to the herd, and when they were all tested ten out of seventeen reacted, and when slaughtered were all found to have tuberculosis to a greater or less extent. Two out of the seventeen were dangerous to human health, and they were all well-bred Jerseys, too. While testing cattle for another firm in this city it is a common practice to condemn from 1 to 20 per cent. as tuberculous. These, too, are animals which show no signs of the disease on physical appearances.

What are we to do with these cattle which react? Is the State to pay for them at a certain value for meat, have them slaughtered, and sell the meat if it be not diseased to any great extent? I say yes, provided the owner will properly disinfect his place and will buy no more cattle without having them tested. To allow cattle which react to go free means that some other dairyman will buy them and place them in his dairy; then, again, supposing that the cattle are tested and react, subsequent inoculation will give a negative result, for a while at any rate, so that the diseased cattle might be passed healthy.

What I say of tuberculosis in cattle does not include all of the dangers of infection. How often do children contract the disease from tuberculous people spitting upon the floor when children are crawling about? How often do people contract the disease by breathing air laden with tuberculous germs? These are questions which I leave for the practitioners of human medicine to determine. Certain it is that if we can have a definite inspection of the milk-supply, and, added to this, a proper observance of rules of decency by infected people, the mortality may be greatly reduced.

Rabies does exist in Maryland, and it exists to a much greater extent than was formerly supposed. Since I became the State Veterinarian I have seen several outbreaks of the disease in cattle bitten by rabid dogs. It is not an imaginary disease by any means. It is not an hysterical disease brought on by fear from having been bitten by an animal supposed to be mad. It is a definite, real disease, and the sooner the public are made aware of it the better it will be for the public and the better it will be for the dogs, and the sooner we will have a dog-law.

It is a mistake to believe that promoters of bench associations are afraid to have the public know this state of affairs, for they are not. It is not the thoroughbred dog that will suffer, but the mongrel cur which runs about our streets and over our pastures; he is the one that does the damage. Just in proportion to the efficacy of laws in different countries does the disease exist. In North Germany the disease is rare ; in Russia it is quite prevalent ; while in France and England it is not uncommon.

Rabies is a distinct disease, propagated only by inoculation. Unless one individual has it, it cannot be spread ; it never arises spontaneously ; when an animal has the disease he runs wild and bites everything in his way unless he be confined. He bites at objects indiscriminately. There is a peculiar, wild look in his eyes ; this stage is followed by the paralytic stage.

Animals bitten show symptoms corresponding to their natures. Sheep and cattle, as a rule, show a tendency to horn and to butt. Horses show a tendency to bite and to kick. I have seen several cases of infection of cattle and sheep. Out of a herd of sixteen cattle one man lost ten from the bite of one dog ; another man lost a number of stock-cattle and many sheep ; still another lost three cows out of four. In two of these cows there was recovery after apparent light attacks of the disease, showing, if this be true, that the disease is not of necessity fatal. These are but a few of the many cases which have come under my notice.

A dog-law, which would tax every dog in the State would soon make the disease a matter of ancient history. Why should not such a law be passed by our Legislature? It has been said that such a law would be trampling upon the poor man ; that it would be legislation for the rich ; not so at all. A law imposing a tax of two or three dollars per year upon each dog would not hurt anybody who could afford to keep a dog. The owners would take better care of them, too. They would be kept at home and looked after; more than that, they would be properly fed, and not half starved, as many of the curs are at the present day. I believe that such a law is perfectly practicable, and I believe it would work well, looked at from a commercial point of view. Why any farmer should be opposed to it is more than I can see, when he knows of the number of sheep he loses in the course of a year from dogs. All licensed dogs should wear a collar, bearing an inscription of the owner's name and the number of the license. All dogs not having this collar should be caught and destroyed. The dogs could be kept somewhat under inspection while awaiting ownership. Dogs with contagious diseases should be quickly separated from the rest, and measures taken for the prevention of the spread of these diseases among the unaffected dogs. As it is now, mongrel dogs are kept in close contact with healthy dogs. Dogs with distemper are placed in the same pen with healthy dogs. At any rate, whatever the method settled upon for its execution, the State should pass a dog-law and should see to it that its penalties be rigidly executed.

As to the disease in horses which has caused and is causing so many deaths, I shall not say much, for the simple reason that not much is to be said. It is a disease which has ordinarily been called cerebro-spinal meningitis, but the queer thing about it is that there are no cerebral or spinal lesions observable. There are no lesions whatever observable upon post-mortem examination. I make this statement not only upon my own observation, but upon the observation of many others. Our worthy President has been with me at autopsies, and he quite agrees with me; so do many more.

Johne, in reviewing Siedamgrotzky and Schlegel's work upon what appears to be the same disease, does not agree with them that there is ever a serious meningitis. Johne, however, finds a diplococcus in the spinal fluid, which, by injection, kills small animals once in a while and makes horses sick, but does not kill them. Acting upon his advice, I obtained from the spinal fluid of a horse which died in Baltimore County a pure culture, which Dr. Stokes found killed rabbits and which we found killed a horse. The same organism which we found in the spinal fluid of the first horse was found in the spinal fluid and organs of the horse and smaller animals inoculated; the symptoms produced are the same as those seen in horses suffering from the disease, though in a much slower form than is generally recognized, namely: rise in temperature, then a fall in temperature; inability to move one hind leg; then the animal gives away behind, and is unable to get up any more. He eats all right up to near the end, but this is only one horse, and not much can be said upon one experiment; I give it for what it is worth, and Dr. Stokes will show you the specimen. You will observe that it is a short bacillus rather than a diplococcus.

The symptoms of this disease in horses are as follows: They are at work as usual; they are either put away at night all right, or they may lag a little in their work. The next morning they are found down and unable to rise; they continue, as a rule, in this way until they die, sometimes living a couple of days and sometimes a week. Sometimes there seems to be a paralysis of the muscles of deglutition, and sometimes they eat and drink for a good while, or nearly up to the time of their death. It has been supposed that weeds of different kinds had much to do with the disease. This is very problematical and has never been proved.

Whatever the disease is, it is extremely fatal. It affects horses in the stable and in the fields, in winter and summer, but probably it is more prevalent in summer than in winter.

